# Semi-field evaluation of electrocuting eave tubes for the control of endophagic mosquitoes in south-east Tanzania

**DOI:** 10.1186/s13071-024-06407-1

**Published:** 2024-08-20

**Authors:** Ruth S. Shirima, Godfrey C. Katusi, Arnold S. Mmbando, Gracious Fanuel, Dimitrios Aslanis, Suhas Kadam, Clement Tshidibi Lonji, Haruna A. Sylvester, Manuel Lluberas, Fredros O. Okumu, Bart G. J. Knols, Emmanuel W. Kaindoa

**Affiliations:** 1 Environmental Health and Ecological Science Department, Ifakara Health Institute, P.O. Box 53, Ifakara, Tanzania; 2 Department of Microbiology and Parasitology, Saint Francis University College of Health and Allied Sciences, Ifakara, Tanzania; 3 Department of Biosciences, Durham University, Durham, DH13LE UK; 4 Wits Research Institute for Malaria, Faculty of Health Science, University of the Witwatersrand, Johannesburg, South Africa; 5 Institute of Biodiversity, Animal Health, and Comparative Medicine, University of Glasgow, Glasgow, G12 8QQ UK; 6 School of Life Science and Bioengineering, The Nelson Mandela African Institution of Science and Technology, P.O. Box 447, Arusha, Tanzania; 7 Robotics From Scratch, Dar Es Salaam, Tanzania; 8 K&S Consulting, Kalkestraat 20, 6669 CP Dodewaard, The Netherlands; 9 Diastec Ltd, 71-75 Shelton Street, Covent Garden, London, WC2H 9JQ UK; 10 College of Agriculture, Dapoli, Maharashtra India; 11 Phoenix University Agwada, Koya/Kana, Nasarawa Nigeria; 12 Mosquito Den LLC, Río Grande, Puerto Rico; 13 Diastec Ltd, 29, Kinsimba Avenue, Ngamiema Municipality, Kinshasa, Congo

**Keywords:** Eave tubes, Malaria, Eave, Electrocuting eave tube

## Abstract

**Background:**

Eave spaces are major entry points through which malaria vectors enter houses. Interventions that target mosquitoes at the eaves have recently been developed. However, most of these interventions are based on insecticides for which resistance has been reported. Here we evaluated the efficacy of mosquito electrocuting eave tubes (MEETs) against *Anopheles gambiae* sensu stricto (*An. gambiae* s.s.) and *Anopheles funestus* s.s. under semi-field conditions.

**Methods:**

Experiments were conducted in two semi-field chambers, each containing one experimental hut. Six electrocuting eave tubes were installed in each hut to assess their impact on laboratory-reared *An. gambiae* s.s. and *An. funestus* s.s.. Each species was assessed separately over 10 nights by releasing 200 unfed females per night into each chamber. One volunteer slept in each hut from 7 p.m. to 5 a.m. Mosquitoes were collected indoors and outdoors using mouth and Prokopack aspirators.

**Results:**

The placement of MEETs significantly reduced the nightly *An. gambiae* s.s. indoor and outdoor biting, by 21.1% and 37.4%, respectively. Indoor-biting *An. funestus* s.s. were reduced by 87.5% while outdoor-biting numbers of *An. funestus* s.s. declined by 10.4%.

**Conclusions:**

MEETs represent a promising tool for controlling mosquitoes at the point of house entry. Further validation of their potential under natural field conditions is necessary. Several advantages over insecticide-based eave tubes are indicated and discussed in this article.

**Graphical Abstract:**

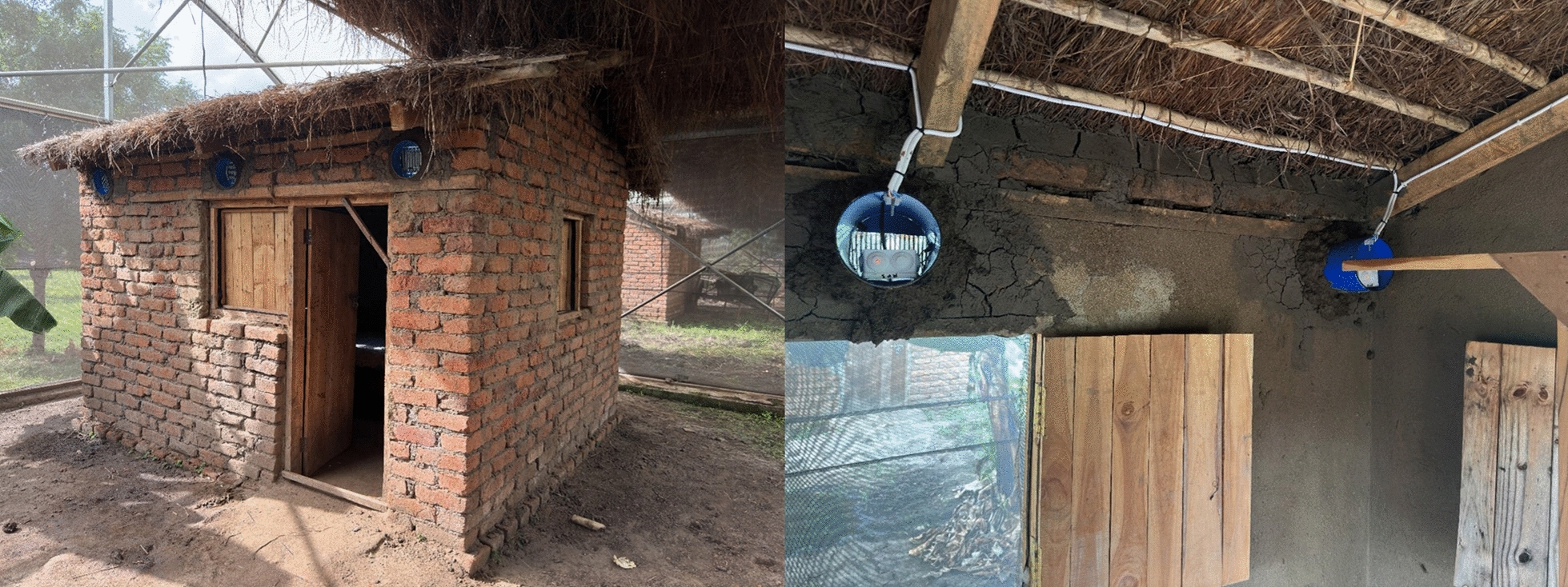

## Background

Malaria remains a major public health concern, with about 249 million reported cases of malaria and 608,000 deaths due to this disease in 2022 [1]. The burden is disproportionately higher in sub-Saharan Africa where about 94% and 95% of all global cases and deaths occurred in 2022, respectively [1]. Despite the pivotal role played by vector control measures such as insecticide-treated nets (ITNs) and indoor residual spraying (IRS) in reducing malaria cases and deaths [2], emerging challenges such as insecticide resistance and shifts in mosquito biting behaviour threaten the efficacy of these interventions [3–6]. The slow-down in the gains in recent years [1] suggests that even with these tools, malaria eradication is unlikely.

In response to the limitations of current vector control strategies, there is an urgent need for innovative and sustainable approaches to malaria prevention. Developing interventions that are not only effective against mosquito vectors but also address the challenges of conventional methods is essential for long-term success in malaria control and ultimately the elimination of malaria [7, 8].

House improvement has been demonstrated to offer protection against malaria [9, 10] and is one of the most preferred interventions for malaria control in southern Tanzania [11, 12]. Several field studies have demonstrated that house design is one of the factors influencing the abundance of indoor malaria vector densities and malaria transmission [13–15]. Simple house modifications can significantly reduce malaria transmission, yet a previous study found that communities in rural Tanzania were unable to improve their houses due to financial constraints and competing household priorities [16]. Poor housing is not only associated with high incidences of malaria transmission but also with other numerous infectious diseases, notably insect-borne diseases such as filariasis and arboviruses, which are major public health and economic concerns in most African countries [9, 13, 14, 17, 18]. In Tanzania, approximately 93% of the population lives in areas at risk of malaria [19], which could be largely preventable by, among other measures, living in proper housing. However, housing is not included in control programmes due to high costs and various challenges [12]. Nonetheless, the major malaria vectors in Africa mostly prefer to feed on humans and bite and rest inside houses [20, 21].

Eave tubes have been introduced as a promising alternative for malaria vector control [22]. The method involves closing the eave spaces while leaving openings for eave tubes, thereby facilitating ventilation in the house while channeling human odour outdoors to attract mosquitoes into the tubes [22]. The tubes are fixed at eave height to target mosquitoes as they attempt to enter houses based on the observation that malaria vectors often use eaves as house entry points [23–25]. As mosquitoes attempt to enter the houses, they come into contact with netting inside the tubes that is treated with insecticides. The insecticide, in powder form, is bound to the netting using electrostatic forces, which increases mosquito exposure and ensures high efficacy even against pyrethroid -resistant mosquitoes [26]. While the use of eave tubes alone, without insecticides, provides a physical barrier against mosquitoes and thus provides protection to the household, the addition of insecticides offers communal protection by effectively killing them [27, 28].

Eave tubes were found to reduce mosquito densities in semi-field studies conducted in Tanzania [28], Kenya [29] and Ivory Coast [30]. In a cluster-randomized trial in Ivory Coast, a combination of house screening and eave tubes significantly reduced malaria incidence [31, 32], as well as the entomological inoculation rate [32]. However, despite the efficacy of this intervention in reducing and killing mosquitoes, inevitably problems with insecticides will arise. Frequent replacement of eave tube inserts with insecticides boosts labour costs and will ultimately cause resistance. Therefore, non-insecticidal approaches are likely to be more sustainable. These can be integrated in long-term vector control strategies without causing resistance buildup in mosquito populations by eliminating selection pressure.

Mosquito electrocuting eave tubes (MEETs) represent a promising alternative in the fight against malaria without relying on insecticides. The MEET utilizes an electrocuting grid installed inside a standard 6-inch (15.24 cm) polyvinyl chloride (PVC) tube that is installed at eave height while other spaces are sealed. In this study, we assessed the efficacy of MEETs against *Anopheles gambiae* sensu stricto (*An. gambiae* s.s.) and *Anopheles funestus* s.s. in a semi-field setting. The results are discussed in the context of utilizing MEETs for and its contribution to sustainable vector control.

## Methods

### Description of the study facility

**Fig. 1:**
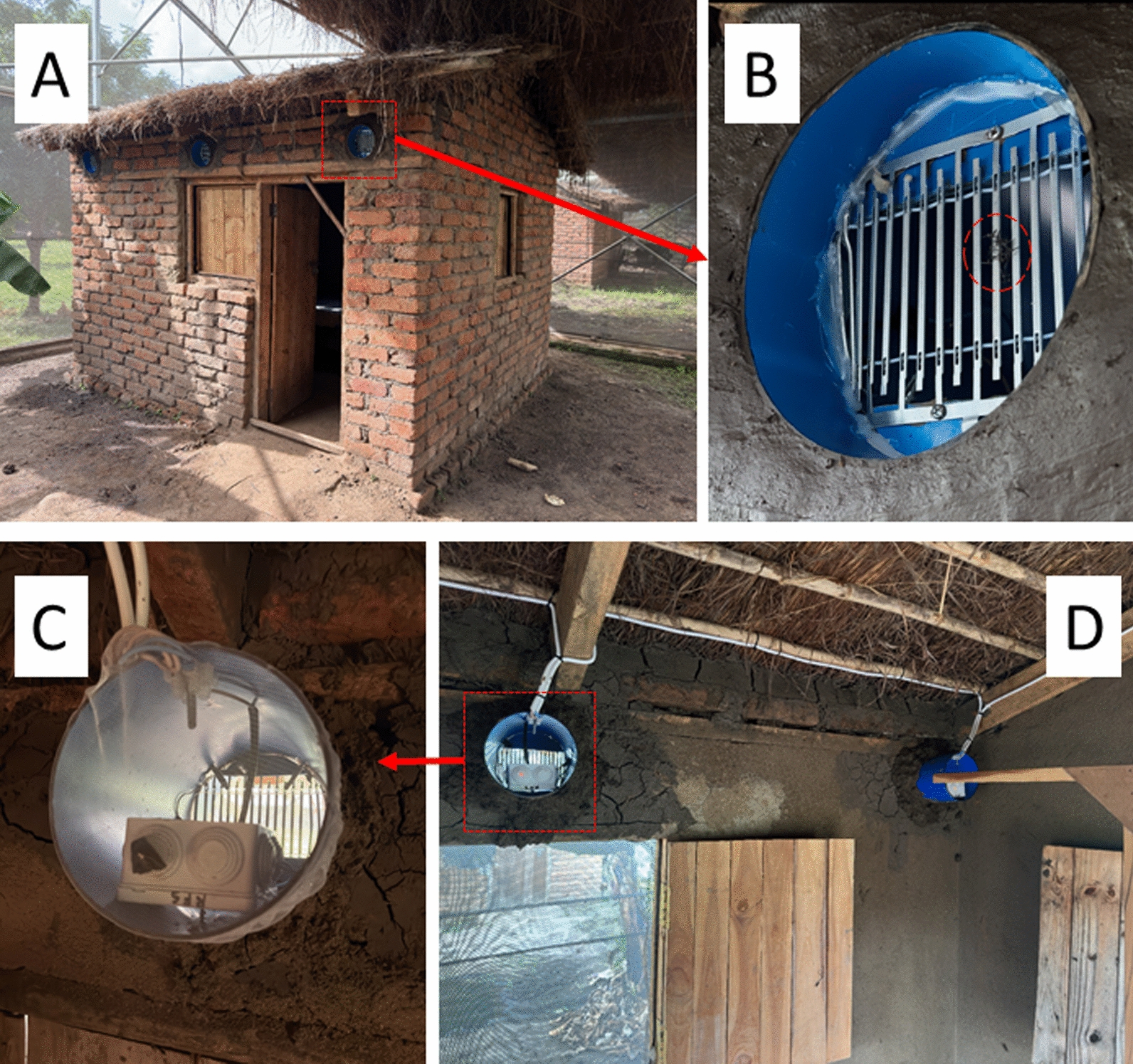
Semi-field set-up for the assessment of the efficacy of mosquito electrocuting eave tubes (MEETs). **A** An experimental hut fitted with 6 MEETs (3 on the front and three at the back). **B** Magnified view of a MEET with a red dashed circle showing some electrocuted mosquitoes. **C** A net fitted in the inner side of the MEET trap. **D** Wiring layout of MEET traps

This study was conducted near Ifakara, south-east Tanzania in the ‘mosquito city’ facility, which is located in Kining’ina village, approximately 6 km north of the town of Ifakara (8.10800°S, 36.66585°E). A semi-field system with a total surface area of 553 m^2^ was utilized (Fig. 1A). The semi-field system comprises of six chambers/compartments, each measuring 9.6 × 9.6 × 4.5 m (length × width × height). Two semi-field chambers within one semi-field system were used. Inside each semi-field chamber, experimental huts measuring 3.1 × 2.7 m were constructed in which the MEETs were installed (Fig. 1B). Each experimental hut had six MEETs. More details on the semi-field systems and experimental huts can be found in previous publications [33–35].

### Mosquito electrocuting eave tubes

The MEETs were made up of standard 6-inch (15.24 cm) PVC tubes, each equipped with an electrocuting grid tightly fitted against the rim of the PVC tube. Grids were removed from commercially available mosquito lamps (model IK GP02; input AC220-240 V 50 Hz; output 800-1000 V through a high-voltage transformer, Tronic, China). The MEETs were serially connected to the mains and were operated permanently by fixing the press button in the ‘on’ mode. Thus, a hut could have all of its grids switched ‘on’ or all of the grids switched ‘off’. To prevent mosquitoes from passing the electrocuting grid and entering the hut, pieces of untreated bednet material were used to cover the tube outlet inside the hut.

### Mosquitoes

Female *An. gambiae* s.s. (Ifakara strain) and *An. funestus* s.s. (FUMOZ strain) reared in the laboratory and maintained at the Ifakara insectaries were used at age 3 to 6 days in the experiments. Larvae were fed on Tetramin fish food (Tetra Werke, Melle, Germany) and maintained at 26–28 °C. Adults were fed ad libitum with a 10% glucose solution, and the insectary was maintained under 12:12-h light/dark conditions.

### Release and recapture of mosquitoes

Each night a total of 200 blood-naive females were released in the semi-field chambers at 7 p.m.−50 mosquitoes in each of the four corners of the semi-field chamber. The mosquitoes had been starved for 6 h prior to the start of the experiment by removing the glucose solution from the insectary. The experiment was conducted for 10 days for each species, with each species released separately.

At 5 a.m., mosquitoes were collected indoors and outdoors using Prokopack and mouth aspirators. The assumption was made that the grids would kill mosquitoes attempting to enter the hut through the PVC tubes. Therefore, the number of live mosquitoes available for collection in the morning was expected to be lower when the grids were ‘on’.

### Efficacy of MEETs against* An. gambiae *s.s. and* An. funestus* s.s.

A comparative study was undertaken in the semi-field system. Two chambers, each with one experimental hut, were used. Six MEETs were fixed in each hut, three on each side. They were installed with the bottom of the pipe aligned with the wall, while the top part was fitted at a slight angle to guide odours coming out of the hut. All other openings (including eaves) were sealed as much as possible with mud so that airflow only passed through the eaves.

Two volunteers slept in each experimental hut from 7 p.m. to 5 a.m.. Each compartment alternated between having all grids turned on for 5 days and all grids turned off for 5 days. Volunteers were rotated randomly to ensure they slept in each hut for 5 days. They were not informed about whether the grids were on or off. Mosquito collection was done by another team, which comprised two volunteers who were also unaware of whether the grids were turned on or off.

### Data analysis

Data were analysed using R software (version 4.1.2) [36]. Data for each species was analysed separately. Descriptive statistics were analysed using the *dplyr* package [37]. Graphics were generated using *ggplot2* [38]. The efficacy of MEETs (percentage reduction in mosquitoes recaptured) was calculated as: $\frac{Mean\, capture \,in \,control-Mean \,capture \,in \,treatment}{Mean \,capture \,in \,control} \times$ 100, where ‘Control’ was the number of mosquitoes recaptured in the ‘MEETs OFF’ chamber, and ‘Treatment’ was the number of mosquitoes recaptured in the ‘MEETs ON’ chamber. The chi-square test of proportions was used to assess whether there was any statistically significant difference between the percentage reduction of mosquito species released, intervention and location. Lastly, to assess the efficacy of MEETs, a generalized linear mixed model (GLMM) was deployed, the number of mosquitoes recaptured was added as a response variable, while the intervention (ON or OFF) was added as a fixed factor. Volunteer identification (ID), chamber ID and experimental day were added as random factors in the models. Model selection for the inclusion of the random effects (volunteers ID, chamber ID and experimental day) was performed using the Akaike information criterion (AIC); the model with the lowest AIC value was considered to be the best model. the odds ratio (OR) was reported along with the respective 95% confidence interval (CI).

## Results

### Mosquito recaptures

A total of 8000 female *Anopheles* mosquitoes were released during the 20-day experiment, of which half were *An. gambiae* s.s., with *An. funestus* s.s. accounting for the other half. A total of 2259 (28% of the released) mosquitoes were recaptured from all chambers, irrespective of the intervention and mosquito species released into the chamber. A similar trend in the recapture rate was observed for both species released. For *An. gambiae* s.s., the recapture rate was lower for the treatment (MEETs ON) chamber than for the control (MEETs OFF) chamber: 28.3% (*n* = 566 of the released mosquitoes recaptured) versus 44% (*n* = 889 of the released mosquitoes recaptured), respectively. Similarly, for *An. funestus* s.s., the recapture rate was lower for the treatment chamber compared with the control chamber: 19.0% (*n* = 379 of the released mosquitoes) versus 21.5% (*n* = 430 of the released mosquitoes).

**Fig. 2:**
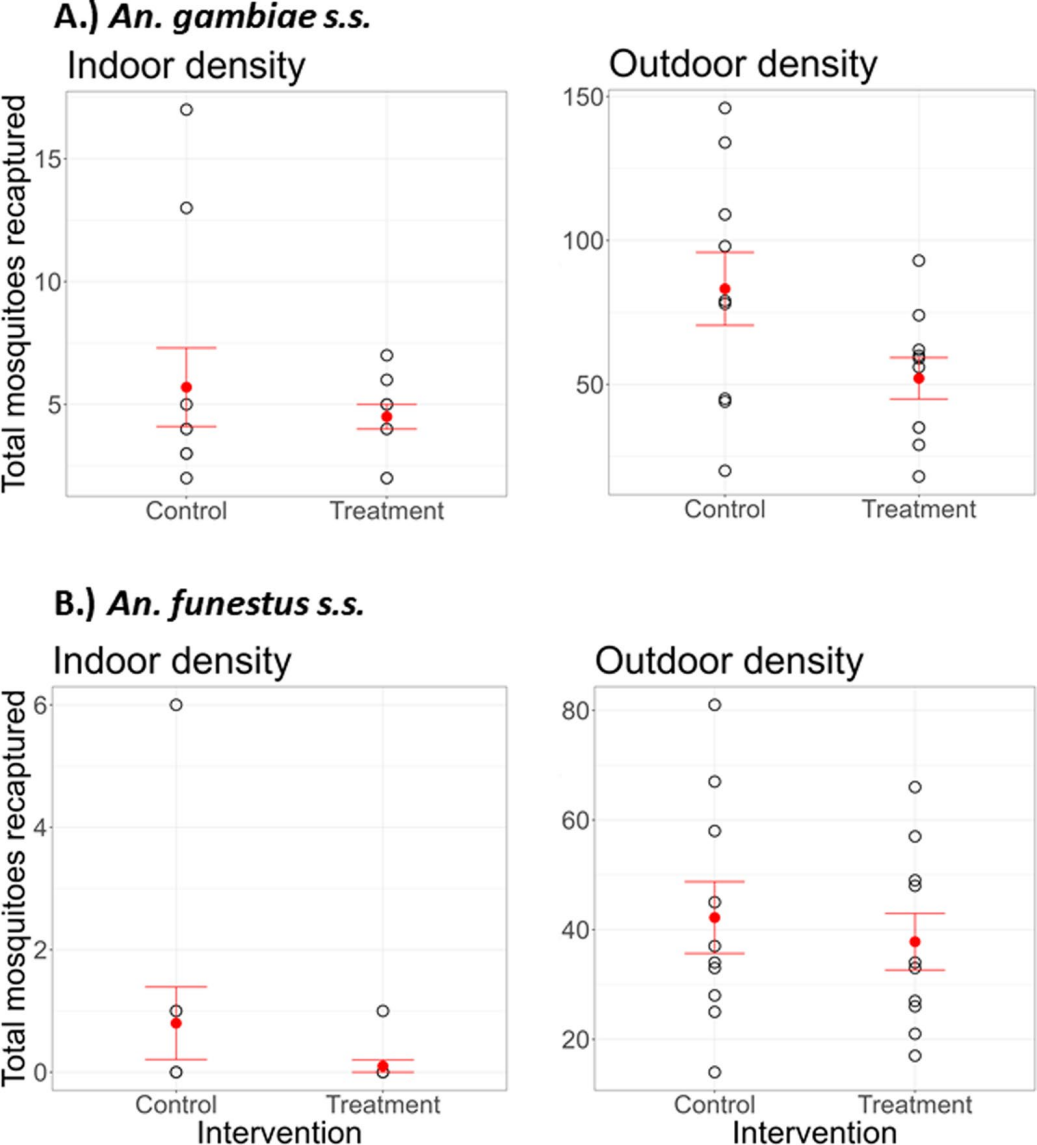
Assessing the efficacy of mosquito electrocuting eave tubes (MEETs). **A** Comparison of different recapture rates of *Anopheles gambiae*s.s. with location. **B** Comparison of different recapture rate of *Anopheles funestus* s.s. with location. Black open circles denote number of mosquitoes recaptured nightly; red circles with error bars denote mean recaptures ± standard error. s.s., Sensu stricto

Despite similar trends in recapture rates, there was a significant difference between the species released and location (*χ*^2^ = 151.06, *df* = 1, *P* < 0.001). The recapture rate of indoor-biting mosquitoes showed a similar catch in the deployed interventions between mosquito species released (*χ*^2^ = 2.47, *df* = 1, *P* = 0.116). The mean (± standard error [SE] indoor recapture rate for *An. gambiae* s.s. was 4.5 ± 0.5 and 5.7 ± 1.6 per day for the treatment and control chambers, respectively. Similarly, the mean indoor recapture rate for *An. funestus* s.s. sensu stricto was 0.1 ± 0.1 and 0.8 ± 0.6 per day for the treatment and control chambers, respectively (Fig. 2).

In comparison, there was a significant difference in the mean outdoor recapture rate across mosquito species following the deployment of the intervention (*χ*^2^ = 15.44, *df* = 1, *P* < 0.001). For *An. gambiae* s.s., the mean (± SE) outdoor recapture rate was 52.1 ± 7.2 and 83.2 ± 12.7 per day for the treatment and control chambers, respectively. Similarly, the mean outdoor recapture rate for *An. funestus* s.s. sensu stricto (*An. funestus* s.s.) was 37.8 ± 5.2 and 42.2 ± 6.5 per day for the treatment and control chambers, respectively (Fig. 2).

### Efficacy of MEETs against* An. gambiae* s.s. and* An. funestus* s.s.

Placement of MEETs reduced the number of female mosquitoes attempting to bite human volunteers in the treatment chamber. The number of indoor- and outdoor-biting *An. gambiae* females was reduced by 21.1% and 37.4% in the treatment chambers (MEETs ON), respectively, relative to the control chambers (MEETs OFF). The observed reduction was statistically significant for both indoor- and outdoor-biting mosquitoes (GLMM: OR = 0.82, *P* < 0.001 and OR = 0.63, *P* < 0.001, respectively; Table 1). Similarly, the number of indoor- and outdoor-biting *An. funestus* s.s. was reduced by 87.5% and 10.42% in the treatment chambers (MEETs ON), respectively, relative to the control chambers. The observed reduction was marginally statistically insignificant for indoor-biting mosquitoes and not significant for outdoor-biting mosquitoes (GLMM: OR = 0.12, *P* = 0.052 and GLMM: OR = 0.88, *P* = 0.086, respectively; Table 1).

**Table 1:** Impact of mosquito electrocuting eave tubes on indoor and outdoor biting densities of *Anopheles gambiae* sensu stricto and *Anopheles funestus* s.s. sensu stricto in the semi-field setting

Species	Indoor			Outdoor		
	OR (95% CI)	Reduction (%)	*P*	OR (95% CI)	Reduction (%)	*P*
	1			1		
*Anopheles gambiae* sensu stricto	0.82 (0.51–1.31)	21.1	< 0.001	0.63 (0.57–0.70)	37.4	< 0.001
*Anopheles funestus* s.s. sensu stricto	0.12 (0.01–1.02)	87.5	0.052	0.88 (0.76–1.02)	10.42	0.086

*CI* Confidence interval, *OR* Odds ratio

## Discussion

Poor house designs in sub-Saharan Africa continue to contribute to malaria transmission [9, 10, 15, 39–41]. One key link in this interaction is the presence of an eave space opening created by household owners to reduce indoor heat stress, but which allows the entry of mosquitoes. Studies have shown that *Anopheles* mosquitoes utilize eave space openings as their main entry point into houses [23–25, 42–44], a behaviour that has formed the basis for several innovative interventions that will kill or repel mosquitoes on their way into the house. Examples of such interventions include screens treated with biological control agents (e.g. entomopathogenic fungi) [45], insecticidal eave curtains [46, 47], repellent-treated eave ribbons [48–52], insecticide-treated eave tubes [22, 27–30], among others. All of these interventions have been shown to effectively control malaria vectors in sub-Saharan Africa. Despite their effectiveness, however, the implementation of these tools is hindered by their dependency on chemicals, to which mosquitoes will inevitably develop resistance. Therefore, complementary non-insecticidal approaches in eave spaces are needed that will provide additional benefits by eliminating selection pressure for insecticide resistance (although it is appreciated that active avoidance of tube entry will ultimately pose a form of behavioural resistance).

The semi-field system in which houses are created to mimic the natural village setting provides a real-life scenario to evaluate the efficacy of MEETs under controlled environments. The main finding of this study was a distinct reduction of recaptures of both *An. gambiae* s.s. and *An. funestus* s.s. within the ‘MEETs-ON’ chambers. Given the innate behaviours of both mosquito species to feed (endophagy) and rest indoors afterwards (endophily), it may be assumed that tube entry occurred while the mosquitoes were host-seeking and that mosquito electrocution upon contact with the grid caused the decline in numbers recaptured. Endophagy is more pronounced for *An. funestus* s.s than for *An. gambiae* s.s., which caused the likely higher reduction of numbers recaptured indoors, with the caveat that numbers were very low indeed.

To the best of our knowledge, this is the first study to directly assess the efficacy of MEETs on mosquitoes. While the objectives were broadly achieved, a number of limitations are noted. First, the study used an indirect measure of reduction in the number of mosquitoes. We could not directly count electrocuted mosquitoes but rather counted recaptured mosquitoes in the chamber. However, this approach has been used in previous studies to assess the effectiveness of eave tubes in Tanzania [28], Kenya [29] and Ivory Coast [30]. Moreover, the Kenya study, in which eave tubes were treated with fluorescent dye that was transferred to mosquitoes upon contact and showed on their bodies after recapture, provided proof of tube entry and contact. Using videography, the same has been shown in open-field studies in Tanzania, where the median contact time of mosquitoes with (treated) netting was 71 s [44]. Second, this study was conducted in a semi-field setting using laboratory-reared mosquitoes; consequently, field trials are required to determine if these efficacies can also be achieved under real-world conditions. A third shortcoming in our study was the large variation in the numbers of recaptured mosquitoes in both the treatment and control chambers during the experimental nights; although the differences were significant, these would likely have been more dramatic if recapture rates had been more consistent, which did occur during previous trials with insecticides in Ifakara [28].

Given that electrocuting grids have been used to study odour-mediated behaviour of mosquitoes [53] and have been considered to be incorporated in traps [54] and as an alternative to human landing catches [55], we assume that approaching mosquitoes were not repelled by the MEETs [56].

Future use of MEETs will likely require their use in areas where houses lack electricity. Given the rapid developments in solar technology, solar conversion efficiency and decreasing costs, it will be relatively easy to run MEETs off-grid in this manner. Moreover, access to solar energy in communities without access to electricity may enhance acceptance of mosquito control tools. Matowo et al. [57] found increased community acceptance of mosquito landing boxes in Tanzania by simply adding solar power to the outdoor devices that rural communities could use for lighting their homes and charging their mobile phones. A trial with odour-baited traps near houses in Kenya achieved the same results through solar panels [58]. Similar approaches could be adopted for scaling up the use of MEETs in similar settings. Considering that the cost of MEETs will be crucial for scalability and accessibility, mass production will be necessary. Fortunately, the electronics of MEETs are already being mass-produced for ‘mosquito rackets’ and are easy to obtain for less than US$1. However, open-field evaluations of MEETs are still necessary in order to obtain a satisfactory prototype for further development.

## Conclusions

MEETs can effectively kill mosquitoes and thus significantly reduce their densities and thereby malaria transmission. MEETs not only provide household protection but may also provide communal protection by mass killing, but this aspect needs to be confirmed in open field trials.

## Data Availability

The data supporting the findings of the study must be available within the article and/or its supplementary materials, or deposited in a publicly available database.

## Abbreviations

AIC: Akaike information criterion
CI: Confidence interval
GLMM: Generalized linear mixed model
IRS: Indoor residual spraying
ITNs: Insecticide-treated nets
MEET: Mosquito electrocuting eave tube
OR: Odds ratio
PVC: Polyvinyl chloride
